# Need and utilization of rehabilitation services in children with cerebral palsy at a referral hospital in Peru: a descriptive study

**DOI:** 10.17843/rpmesp.2025.423.15834

**Published:** 2026-06-30

**Authors:** Roger De la Cerna-Luna, Alexander G. Acevedo-Cahuana, Brenda Galindo-Yllu, Elvira G. Zamora-Huaringa, Mabel Ramirez-Chipana, Pilar Merino-Orbegoso

**Affiliations:** 1 Servicio de Rehabilitación Pediátrica, Hospital Nacional Edgardo Rebagliati Martins, EsSalud, Lima, Peru.; 2 Escuela de Posgrado, Universidad Peruana Cayetano Heredia, Lima, Peru.; 3 Unidad de Investigación Clínica y Epidemiológica, Escuela de Medicina, Universidad Peruana Unión, Lima, Peru.; 4 Evidence Generation and Epidemiological Surveillance Unit, Scientia Clinical and Epidemiological Research Institute, Trujillo, Peru.; 5 Servicio de Medicina Física y Rehabilitación, Hospital Central de la Policía Nacional del Perú Luis N. Sáenz, Dirección de Sanidad Policial, Lima, Peru.

**Keywords:** Cerebral palsy, Muscle Spasticity, Botulinum Toxins, Hip Dislocation, Physical Medicine and Rehabilitation, Peru

## Abstract

With the objective of describing the characteristics and comparing the need with the previous utilization of rehabilitation services in children with cerebral palsy at a referral hospital in Peru, a descriptive study was conducted through the review of medical records of 86 patients treated during 2024. It was found that 41.8% did not attend any educational institution and 44.2% did not have a disability certificate; among those who had one, the mean time to obtain it was 4.9 years. Less than half had a pelvic radiograph, despite hip dislocation being a frequent comorbidity. Although the majority required rehabilitation therapies, their coverage was limited, especially in occupational therapy and speech/swallowing therapy. Likewise, more than half of those who required botulinum toxin had not received it. These findings demonstrate significant discrepancies between the need and previous utilization of rehabilitation services in this population.

## INTRODUCTION

Cerebral palsy (CP) is a heterogeneous neurodevelopmental disorder that originates in early life stages, due to a non-progressive brain lesion or alteration, and that compromises motor function and frequently other functional areas ^1^. It is the main cause of childhood disability worldwide, and its main risk factors include prematurity, low birth weight, hypoxia, and infections ^2^. It is associated with epilepsy, intellectual disability, and musculoskeletal, sensory, speech, and swallowing alterations, generating significant functional limitations and a lower quality of life ^2^^,^^3^. Its prevalence in low- and middle-income countries is estimated at up to 3.4 per 1000 live births ^4^, and in Peru, the only estimate reported in 1993 was 5.2 per 1000 live births ^5^.

CP represents a relevant public health problem; indicators such as prevalence, motor severity, and comorbidities allow evaluating the impact of complications, the effectiveness of healthcare systems, guiding health policies, and monitoring access to health services ^6^. According to the Global Burden of Disease (GBD) Study 2019, CP generated a high burden of years lived with disability in children under five years of age, especially in low- and middle-income countries (LMICs) ^7^. In Europe, gaps in access to health services for people with CP persist despite having universal public systems ^8^, and in Peru, a significant educational, social, and economic impact has been reported in children with CP, as well as difficulties in accessing health services, which prevents early detection and timely management of comorbidities ^6^^,^^9^.

However, no studies have been identified that evaluate in detail the availability and utilization of rehabilitation services in children with CP in Peru, including physiatric interventions for spasticity management, one of its most disabling complications. Therefore, the objective of this study is to describe the clinical and functional characteristics, and compare the need with the prior utilization of rehabilitation services in children with CP treated at the Pediatric Rehabilitation Service of Hospital Nacional Edgardo Rebagliati Martins (SRP-HNERM) in Lima, Peru, during 2024.

**KEY MESSAGES**

Motivation for the study. Children with cerebral palsy (CP) in Peru face multiple care needs; however, there is scarce evidence about their rehabilitation needs and the gaps between these and the utilization of available services.

Main findings. Significant gaps were evidenced between the need for and utilization of rehabilitation services in children with CP, particularly in occupational therapy, speech/swallowing therapy, and spasticity management. In addition, the absence of a disability certificate, limited radiographic hip surveillance, and high functional and clinical impairment were frequent. Public health implications. These findings highlight the urgent need to improve access to timely interventions to optimize outcomes and quality of life in children with CP in Peru.

## THE STUDY

A descriptive study was conducted using information from the electronic medical records of children with CP treated at the SRP-HNERM. The sample was comprised of all patients treated between January 1 and December 31, 2024, who met the selection criteria. Underage patients treated during said period were included, and those with incomplete electronic medical records were excluded.

It was decided to study children with CP treated at the SRP-HNERM because this service applies a standardized protocol for the evaluation of all its patients ^10^, which allowed for a more comprehensive characterization of this population. During 2024, the SRP-HNERM had only four physiatrists, two physical therapists, one occupational therapist, and one speech/swallowing therapist for the outpatient and hospital care of children with CP.

After obtaining approval from the ethics committee, a list of all children with CP seen in medical consultation at the SRP-HNERM during the year 2024 was requested. Subsequently, 86 electronic medical records were manually reviewed to extract the relevant information into a Microsoft Excel spreadsheet.

Variables were considered regarding general characteristics, functional and clinical characteristics, related to the need (according to the physiatrist's evaluation in the outpatient clinic, based on the clinical history, physical examination, and the patient's functional performance) and the previous utilization of rehabilitation services (history of receiving physiatric interventions for spasticity management and rehabilitation therapies), use of orthoses or assistive devices, and pharmacological treatment, which are detailed in a variable operationalization table (supplementary material).

The data collected in the spreadsheet were imported into Stata software version 18.0 (StataCorp LLC, College Station, Texas, United States). Numerical variables were reported as means and standard deviations or medians and interquartile ranges, based on their normality evaluated using the Shapiro-Wilk test. Categorical variables were presented as absolute and relative frequencies.

This study was approved by the Institutional Research Ethics Committee of HNERM (Ethical Qualification Certificate AUT No. 095-CE-GHNERM-GRPR-ESSALUD-2024). All information collected was kept strictly confidential and was used exclusively for the purposes of the study.

## FINDINGS

Eighty-six children with CP were evaluated. The median age was nine years. Most were males (57%), resided in Lima (74.4%), were continuing patients (72.1%), were underweight (44.2%), and had no history of prematurity (61.6%). 47.7% presented with severe/profound intellectual disability, 18.6% had no intellectual quotient evaluation, and 41.8% did not attend any educational institution. 44.2% did not have a disability certificate and, among those who had one, 46.5% were classified with severe gravity, with a mean time for its obtainment of 4.86 years from the diagnosis of CP (Table 1).

**Table 1:** General characteristics of children with cerebral palsy attended at the Pediatric Rehabilitation Service of the Hospital Nacional Edgardo Rebagliati Martins in Lima, Peru, during 2024 (n=86).

Variable		n (%)
Age in years		9 (6-11)^a^
Sex		
	Male	49 (57.0)
	Female	37 (43.0)
Address		
	Lima	64 (74.4)
	Outside Lima	22 (25.6)
Type of patient		
	Continuator	62 (72.1)
	New	24 (27.9)
Body mass index		
	Normal	40 (46.5)
	Low weight	38 (44.2)
	Overweight	8 (9.3)
History of prematurity		
	No	53 (61.6)
	Moderate to late preterm	13 (15.1)
	Very premature	5 (5.8)
	Extremely premature	15 (17.5)
Intellectual disability		
	Severe/profound	41 (47.7)
	Mild	5 (5.8)
	No	24 (27.9)
	Without evaluation	16 (18.6)
Education		
	None	36 (41.8)
	Basic regular	35 (40.7)
	CEBE	14 (16.3)
	PRITE	1 (1.2)
Had a disability certificate		48 (55.8)
	Mild severity	3 (3.5)
	Moderate severity	5 (5.8)
	Severe severity	40 (46.5)
Time in months without disability certificate since CP diagnosis (n=38)		4.86 (3.22)^b^

CEBE: Special Basic Education Center, PRITE: Early Intervention Program, CP: cerebral palsy.

a Median (interquartile range), b mean (standard deviation).

**Figure 1:**
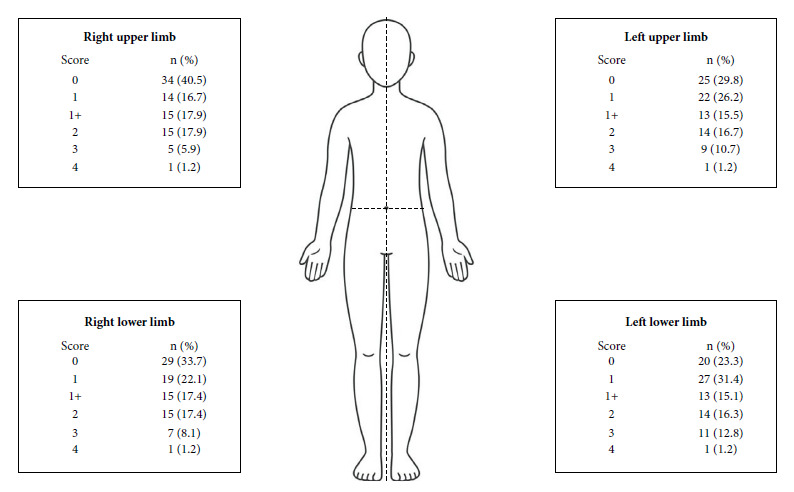
Muscle tone (mean score on the Modified Ashworth Scale) per limb in children with cerebral palsy (n=86).

Most presented spastic hemiparesis (37.2%) or spastic quadriparesis (27.9%), as well as high levels of functional disability (Gross Motor Function Classification System [GMFCS] V, Manual Ability Classification System [MACS] V, Eating and Drinking Ability Classification System [EDACS] III-IV). The most frequent comorbidities were epilepsy (53.5%) and hip dislocation (23.3%). Only 8.1% had gastrostomy and 47.7% had a pelvic X-ray. Most presented loss of passive range of joint movement (ROM) in one or more joints (73.3%) (table 2). In relation to muscle tone, the left hemibody was the most affected (figure 1).

**Table 2:** Functional and clinical characteristics of children with cerebral palsy attended at the Pediatric Rehabilitation Service of the Hospital Nacional Edgardo Rebagliati Martins in Lima, Peru, during 2024 (n=86).

Characteristic		n (%)
Classification by subtypes		
	Spastic hemiparesis	32 (37.2)
	Spastic quadriparesis	24 (27.9)
	Spastic paraparesis	10 (11.6)
	Spastic quadriplegia	8 (9.3)
	Spastic paraplegia	1 (1.2)
	Other	11 (12.8)
GMFCS		
	V	36 (41.8)
	IV	8 (9.3)
	III	4 (4.7)
	II	27 (31.4)
	I	11 (12.8)
MACS		
	V	26 (30.2)
	IV	14 (16.3)
	III	11 (12.8)
	II	20 (23.3)
	I	15 (17.4)
EDACS		
	V	9 (10.5)
	IV	21 (24.4)
	III	21 (24.4)
	II	16 (18.6)
	I	19 (22.1)
Comorbidities		
	Epilepsy	46 (53.5)
	Hip dislocation	20 (23.3)
	Hydrocephalus	14 (16.3)
	Equinovarus foot	12 (13.9)
	Scoliosis	11 (12.8)
	Respiratory disease	10 (11.6)
Had gastrostomy		7 (8.1)
Had a pelvis X-ray		41 (47.7)
Had loss of passive RAM in one or more joints		63 (73.3)

GMFCS: Gross Motor Function Classification System, MACS: Manual Ability Classification System, EDACS: Eating and Drinking Ability Classification System, RAM: range of motion.

Regarding the need for and prior use of rehabilitation services, although 55.8% needed botulinum toxin type A (BoNT-A) for spasticity management, the majority had not received it previously (58.3%). 68.6% required diagnostic nerve blocks. Almost all required physical therapy (PT) (99.8%), occupational therapy (OT) (90.7%), and speech/swallowing therapy (ST) (75.6%); however, only 54.1% had previously received PT, while the majority had not received OT (85.9%) or ST (81.5%). Likewise, 19.8% interrupted their rehabilitation therapies for one to four years due to the COVID-19 pandemic, with a mean interruption time of 2.4 years (table 3).

**Table 3:** Need and utilization of rehabilitation services, use of orthoses or assistive devices, and pharmacological treatment of children with cerebral palsy attended at the Pediatric Rehabilitation Service of the Hospital Nacional Edgardo Rebagliati Martins in Lima, Peru, during 2024 (n=86).

Characteristic		n (%)
Need for DNB for spasticity management		59 (68.6)
Need for BoNT-A for management of spasticity (n=48)		
	Previously received BoNT-A	20 (41.7)
	Never received BoNT-A	28 (58.3)
Need for PT (n=85)		
	Previously received PT	46 (54.1)
	Never received PT	39 (45.9)
Need for OT (n=78)		
	Received previously OT	11 (14.1)
	Never received OT	67 (85.9)
Need for ST (n=65)		
	Previously received ST	12 (18.5)
	Never received ST	53 (81.5)
Time in years elapsed without rehabilitation therapies (n=17)		
	1 year	2 (11.7)
	2 years	7 (41.2)
	3 years	7 (41.2)
	4 years	1 (5.9)
Mean time in years without rehabilitation therapies^a^		2.41 (0.79)^b^
Use of orthoses and assistive devices		74 (86.0)
	Ankle-foot orthosis	60 (69.8)
	Wheelchair/adapted car	40 (46.5)
	Long knee orthosis	30 (34.9)
	Wrist-hand splint	27 (31.4)
	Static elbow orthosis	18 (20.9)
	Semirigid thoracolumbar orthosis	11 (12.8)
	Walker	5 (5.8)
Use of anticonvulsants		46 (53.5)
	One	21 (24.4)
	Two	14 (16.3)
	Three	11 (12.8)
Use of baclofen		26 (30.2)
Use of bronchodilators		9 (10.5)
Use of cannabinoids		3 (3.5)

DNB: diagnostic nerve block; BoNT-A: botulinum toxin type A; PT: physical therapy; OT: occupational therapy; ST: speech/swallowing therapy.

a Time elapsed without rehabilitation therapies due to the COVID-19 pandemic, since the last therapy session, ^b^ mean (standard deviation)

86% used orthoses or assistive devices, the most frequent being ankle-foot orthoses (AFO) (69.8%), wheelchair or adapted car (46.5%), and long knee orthosis (34.9%). Regarding pharmacological treatment, 53.5% used anticonvulsants, and 30.2% baclofen (table 3).

## DISCUSSION

Most children with CP were underweight. 47.7% presented with severe/profound intellectual disability and 41.8% did not attend any educational institution. Slightly less than half did not have a disability certificate and, among those who had it, the average time to obtain it was 4.86 years. Hip dislocation was one of the most frequent comorbidities, but less than half had a pelvic X-ray. 45.9%, 85.9%, and 81.5% of those requiring PT, OT, and SLT, respectively, had never received them, despite the high frequency of moderate to severe limitations in gross motor function (GMFCS V), manipulation (MACS V), and swallowing (EDACS III-IV), including gastrostomy use.

A systematic review published in 2022 reported a prevalence of malnutrition close to 40% in children with CP, associated with food scarcity and socioeconomic and age factors ^11^. The prevalence observed in this study could be explained by findings from the Global CP Registry in LMICs, which reported higher rates of malnutrition associated with lower maternal education and greater motor impairment (GMFCS III-V) ^12^. It should be noted that weight/age is the most used anthropometric measure to assess nutritional status ^11^; however, BMI was used in the SRP-HNERM.

The GBD 2019 reported a high prevalence of intellectual disability in patients with CP, and highlighted the importance of early identification and intervention, especially in LMICs, to improve school readiness and promote inclusive education ^7^. In Peru, since 2024, a law promotes the training of health professionals and teachers in inclusive education for children with CP ^13^. A cohort study in Australia found that only 30% of children with CP had normal scores on cognitive tests, with most having hemiplegia or hemiparesis ^14^, which aligns with the findings of this study.

In Peru, the General Law on Persons with Disabilities establishes that certification accredits the condition of a person with a disability and grants social and labor benefits ^15^; however, this study found a prolonged mean time for its obtainment, which could be related to the mean age of PC diagnosis previously reported (4.1 years) ^5^. Studies in the region have identified barriers to disability certification, such as lack of medical knowledge, technological limitations, and logistical difficulties, including limited internet access and residence in rural areas ^16^.

Established hip surveillance programs for children with CP recommend clinical and radiographic evaluation between the first and second year or from diagnosis, for early detection and treatment ^17^. Patients with GMFCS V have up to a 90% risk of hip dislocation, a condition that significantly affects function and quality of life by limiting sitting, mobility, and daily activities, and constitutes a frequent cause of pain ^17^.

A cross-sectional study conducted in 2023 in children with CP mostly treated in public hospitals in Saudi Arabia found that 96.4% had received PT, but that 65.9% and 85.7% had not received OT and ST, respectively ^18^. A cross-sectional study conducted in 2025 in children with CP treated in rehabilitation centers in Argentina found that all had received at least one weekly hour of rehabilitation therapies ^19^. The differences with the present study could be explained by the low number of physiatrists and rehabilitation therapists at the SRP-HNERM for the care of this population. Additionally, a 2016 study in Peruvian children with CP found a median waiting time of two months to receive PT ^7^. People with disabilities also face other barriers to accessing rehabilitation services, such as families' lack of knowledge about their importance and limitations in access to transportation ^19^.

This study presents some limitations that should be considered when interpreting its results. Firstly, being a retrospective study based on medical records, there is the possibility of registration errors and incomplete information for some variables not systematized in usual clinical practice. Secondly, the study only includes children with CP treated at the SRP-HNERM, which introduces a significant selection bias, since this population is not representative of all children with CP in Peru, particularly those treated in Ministry of Health (MINSA) establishments in Peru or those who do not access any level of care. In these groups, the utilization of rehabilitation services could be lower or even nonexistent, so the findings could underestimate or overestimate certain gaps in other care settings. Finally, due to its cross-sectional and unicentric design, the results do not allow for establishing causal inferences nor are they generalizable to the total population of children with CP in the country. However, this study provides detailed evidence from a group treated at a referral center, which allows for describing patterns of need and utilization in an environment with relative availability of rehabilitation services.

In conclusion, a significant discrepancy was evidenced between the need and the prior utilization of rehabilitation services in the evaluated children with CP, especially in occupational therapy, speech/swallowing therapy, and spasticity management, as well as in hip radiographic surveillance and the obtaining of the disability certificate. This situation occurred in the context of a population with a high burden of functional impairment and comorbidities, including low weight, severe/profound intellectual disability, and significant limitations in motor and feeding function. Together, these findings provide relevant descriptive evidence for understanding the gap between the need and the utilization of rehabilitation services in this clinical context.
