# Supplementary material for: Need and utilization of rehabilitation services in children with cerebral palsy at a referral hospital in Peru: a descriptive study
Source: Rev Peru Med Exp Salud Publica. 2026 Jun 30;43(2):244–50. doi: 10.17843/rpmesp.2025.423.15834 (PMC13456931; doi:10.17843/rpmesp.2025.423.15834)
Supplement: Supplementary file 2 [file rpmesp-43-02-15834-s001.docx]

**Material Suplementario**

**Tabla de operacionalización de variables.**

| **Variable** | **Definición operacional** | **Tipo de variable** | **Escala de medición** |
| --- | --- | --- | --- |
| Edad | Años cumplidos al momento de la atención | Cuantitativa | Razón |
| Sexo | Masculino o femenino | Cualitativa | Nominal |
| Residencia | Lugar de residencia (Lima, Fuera de Lima) | Cualitativa | Nominal |
| Tipo de paciente | Nuevo, Continuador | Cualitativa | Nominal |
| Índice de masa corporal | Clasificación nutricional (kg/m^2^) (Normal, Bajo peso, Sobrepeso) | Cualitativa | Ordinal |
| Prematuridad | Antecedente de prematuridad (No, Prematuro moderado a tardío, Muy prematuro, Prematuro extremo) | Cualitativa | Ordinal |
| Discapacidad intelectual | Grado de discapacidad intelectual (No, Leve, Moderada, Severa, Profunda, Sin evaluación) | Cualitativa | Ordinal |
| Institución educativa | Tipo de institución educativa (Ninguna, Básica Regular, Centro de Educación Básica Especial [CEBE], Programa de Intervención Temprana [PRITE]) | Cualitativa | Nominal |
| Certificado de discapacidad | Tenencia de certificado de discapacidad (Sí, No) | Cualitativa | Nominal |
| Gravedad de la discapacidad | Gravedad de la discapacidad (Leve, Moderada, Severa) | Cualitativa | Ordinal |
| Tiempo para la obtención del certificado de discapacidad | Años transcurridos desde el diagnóstico de parálisis cerebral hasta la obtención del certificado de discapacidad | Cuantitativa | Razón |
| Subtipo de parálisis cerebral | Clasificación clínica/topográfica de la parálisis cerebral (Hemiparesia espástica, Cuadriparesia espástica, Paraparesia espástica, Otra) | Cualitativa | Nominal |
| Clasificación funcional de la motricidad gruesa | Sistema de Clasificación de la Función Motora Gruesa (GMFCS) (I, II, III, IV, V) | Cualitativa | Ordinal |
| Clasificación funcional de la motricidad fina | Sistema de Clasificación de la Habilidad Manual (MACS) (I, II, III, IV, V) | Cualitativa | Ordinal |
| Clasificación funcional de la alimentación | Sistema de Clasificación de la Habilidad para Comer y Beber (EDACS) (I, II, III, IV, V) | Cualitativa | Ordinal |
| Comorbilidades | Presencia de comorbilidades (Epilepsia, Luxación de cadera, Hidrocefalia, Pie equinovaro, Escoliosis, Enfermedad respiratoria) | Cualitativa | Nominal |
| Gastrostomía | Portador de gastrostomía (Sí, No) | Cualitativa | Nominal |
| Radiografía de pelvis | Presencia de radiografía de pelvis (Sí, No) | Cualitativa | Nominal |
| Rango articular de movimiento (RAM) | Grados de RAM medidos por goniometría | Cuantitativa | Razón |
| Tono muscular | Tono muscular según puntaje en la Escala Modificada de Ashworth (MAS) (0, 1, 1+, 2, 3, 4) | Cualitativa | Ordinal |
| Necesidad de bloqueo nervioso diagnóstico (DNB) | Requería bloqueo nervioso diagnóstico para manejo de la espasticidad (según evaluación del fisiatra en la consulta externa, basada en la historia clínica, el examen físico y el desempeño funcional del paciente) (Sí, No) | Cualitativa | Nominal |
| Necesidad de toxina botulínica (BoNT-A) | Requería BoNT-A para manejo de la espasticidad (según evaluación del fisiatra en la consulta externa, basada en la historia clínica, el examen físico y el desempeño funcional del paciente) (Sí, No) | Cualitativa | Nominal |
| Recepción previa de BoNT-A | Antecedente de haber recibido BoNT-A (Sí, No) | Cualitativa | Nominal |
| Necesidad de terapia física (TF) | Requería TF (según evaluación del fisiatra en la consulta externa, basada en la historia clínica, el examen físico y el desempeño funcional del paciente) (Sí, No) | Cualitativa | Nominal |
| Recepción previa de TF | Antecedente de haber recibido TF (Sí, No) | Cualitativa | Nominal |
| Necesidad de terapia ocupacional (TO) | Requería TO (según evaluación del fisiatra en la consulta externa, basada en la historia clínica, el examen físico y el desempeño funcional del paciente) (Sí, No) | Cualitativa | Nominal |
| Recepción previa de TO | Antecedente de haber recibido TO (Sí, No) | Cualitativa | Nominal |
| Necesidad de terapia de lenguaje/deglución (TL) | Requería TL (según evaluación del fisiatra en la consulta externa, basada en la historia clínica, el examen físico y el desempeño funcional del paciente) (Sí, No) | Cualitativa | Nominal |
| Recepción previa de TL | Antecedente de haber recibido TL (Sí, No) | Cualitativa | Nominal |
| Tiempo transcurrido sin terapias de rehabilitación debido a la pandemia de COVID-19 | Años transcurridos de interrupción terapéutica debido a la pandemia de COVID-19, desde la última sesión de terapia de rehabilitación (TF, TO o TL) | Cuantitativa | Razón |
| Uso de ortesis | Uso de ortesis (Ortesis tobillo-pie, Ortesis larga de rodilla, Ortesis estática de codo, Ortesis toracolumbar semirrígida) | Cualitativa | Nominal |
| Uso de dispositivos de asistencia | Uso de dispositivos de asistencia (Silla de ruedas o Coche adaptado, Andador) | Cualitativa | Nominal |
| Uso de anticonvulsivantes | Número de fármacos anticonvulsivante usados (Uno, Dos, Tres,) | Cualitativa | Ordinal |
| Uso de otros fármacos | Uso de otros fármacos (Baclofeno, Broncodilatadores, Cannabinoides) | Cualitativa | Nominal |
