# Supplementary material for: Need and utilization of rehabilitation services in children with cerebral palsy at a referral hospital in Peru: a descriptive study
Source: Rev Peru Med Exp Salud Publica. 2026 Jun 30;43(2):244–50. doi: 10.17843/rpmesp.2025.423.15834 (PMC13456931; doi:10.17843/rpmesp.2025.423.15834)
Supplement: Translation

# Translation: Necesidad y utilización de servicios de rehabilitación en niños con parálisis cerebral en un hospital de referencia en Perú: un estudio descriptivo

**Authors:** Roger De la Cerna-Luna, Alexander G. Acevedo-Cahuana, Brenda Galindo-Yllu, Elvira G. Zamora-Huaringa, Mabel Ramirez-Chipana, Pilar Merino-Orbegoso

**Affiliations:** 1 Servicio de Rehabilitación Pediátrica, Hospital Nacional Edgardo Rebagliati Martins, EsSalud, Lima, Perú.; 2 Escuela de Posgrado, Universidad Peruana Cayetano Heredia, Lima, Perú.; 3 Unidad de Investigación Clínica y Epidemiológica, Escuela de Medicina, Universidad Peruana Unión, Lima, Perú.; 4 Evidence Generation and Epidemiological Surveillance Unit, Scientia Clinical and Epidemiological Research Institute, Trujillo, Perú.; 5 Servicio de Medicina Física y Rehabilitación, Hospital Central de la Policía Nacional del Perú Luis N. Sáenz, Dirección de Sanidad Policial, Lima, Perú.

**Keywords:** Parálisis cerebral, Espasticidad Muscular, Toxinas Botulínicas, Luxación de la Cadera, Medicina física y Rehabilitación, Perú

## Abstract

Con el objetivo de describir las características y comparar la necesidad con la utilización previa de servicios de rehabilitación en niños con parálisis cerebral en un hospital de referencia en Perú, se realizó un estudio descriptivo mediante la revisión de historias clínicas de 86 pacientes atendidos durante el 2024. Se encontró que el 41,8% no asistía a ninguna institución educativa y el 44,2% no contaba con certificado de discapacidad; entre quienes lo tenían, la media de tiempo para su obtención fue de 4,9 años. Menos de la mitad contaba con radiografía de pelvis, a pesar de que la luxación de cadera fue una comorbilidad frecuente. Aunque la mayoría requería terapias de rehabilitación, su cobertura fue limitada, especialmente en terapia ocupacional y de lenguaje/deglución. Asimismo, más de la mitad de quienes requerían toxina botulínica no la habían recibido. Estos hallazgos evidencian importantes discrepancias entre la necesidad y la utilización previa de servicios de rehabilitación en esta población.

## INTRODUCCIÓN

La parálisis cerebral (PC) es un trastorno heterogéneo del neurodesarrollo que se origina en etapas tempranas de la vida, por una lesión o alteración cerebral no progresiva, y que compromete la función motora y frecuentemente otras áreas funcionales ^1^. Es la principal causa de discapacidad infantil a nivel mundial, y sus principales factores de riesgo incluyen prematuridad, bajo peso al nacer, hipoxia e infecciones ^2^. Se asocia con epilepsia, discapacidad intelectual y alteraciones musculoesqueléticas, sensoriales, del habla y de la deglución, generando limitaciones funcionales significativas y una menor calidad de vida ^2^^,^^3^. Su prevalencia en países de ingresos bajos y medianos se estima hasta en 3,4 por cada 1000 nacidos vivos ^4^, y en el Perú, la única estimación reportada en 1993 fue de 5,2 por cada 1000 nacidos vivos ^5^.

La PC representa un problema relevante de salud pública; indicadores como la prevalencia, la severidad motora y las comorbilidades permiten evaluar el impacto de las complicaciones, la efectividad de los sistemas de salud, orientar políticas sanitarias y monitorear el acceso a servicios de salud ^6^. Según el Estudio de Carga Global de Enfermedad (GBD) 2019, la PC generaba una alta carga de años vividos con discapacidad en menores de cinco años, especialmente en países de ingresos bajos y medianos (LMICs) ^7^. En Europa persisten brechas de acceso a servicios de salud para personas con PC pese a contar con sistemas públicos universales ^8^, y en Perú se ha reportado una importante repercusión educativa, social y económica en niños con PC, así como dificultades para acceder a servicios de salud, lo que impide una detección temprana y un manejo oportuno de las comorbilidades ^6^^,^^9^.

Sin embargo, no se han identificado estudios que evalúen en detalle la disponibilidad y utilización de los servicios de rehabilitación en niños con PC en el Perú, incluyendo las intervenciones fisiátricas para el manejo de la espasticidad, una de sus complicaciones más discapacitantes. Por ello, el objetivo de este estudio es describir las características clínicas y funcionales, y comparar la necesidad con la utilización previa de servicios de rehabilitación en niños con PC atendidos en el Servicio de Rehabilitación Pediátrica del Hospital Nacional Edgardo Rebagliati Martins (SRP-HNERM) en Lima, Perú, durante el 2024.

**MENSAJES CLAVE**

Motivación para realizar el estudio. Los niños con parálisis cerebral (PC) en Perú enfrentan múltiples necesidades de atención; sin embargo, existe escasa evidencia sobre sus necesidades de rehabilitación y las brechas entre estas y la utilización de los servicios disponibles.

Principales hallazgos. Se evidenciaron importantes brechas entre la necesidad y la utilización de servicios de rehabilitación en niños con PC, particularmente en terapia ocupacional, terapia de lenguaje/deglución y manejo de la espasticidad. Además, fueron frecuentes la ausencia de certificado de discapacidad, la limitada vigilancia radiográfica de cadera y el elevado compromiso funcional y clínico. Implicancias en salud pública. Estos hallazgos resaltan la necesidad urgente de mejorar el acceso a intervenciones oportunas para optimizar los resultados y la calidad de vida en los niños con PC de Perú.

## EL ESTUDIO

Se realizó un estudio descriptivo utilizando información de las historias clínicas electrónicas de niños con PC atendidos en el SRP-HNERM. La muestra estuvo conformada por todos los pacientes atendidos entre el 1 de enero y el 31 de diciembre de 2024, que cumplieron con los criterios de selección. Se incluyeron pacientes menores de edad atendidos durante dicho período y se excluyeron aquellos con historias clínicas electrónicas incompletas.

Se decidió estudiar a los niños con PC atendidos en el SRP-HNERM debido a que este servicio aplica un protocolo estandarizado para la evaluación de todos sus pacientes ^10^, lo que permitió una caracterización más integral de esta población. Durante el 2024, el SRP-HNERM contó con solo cuatro fisiatras, dos fisioterapeutas, una terapeuta ocupacional, y un terapeuta de lenguaje/deglución para la atención ambulatoria y hospitalaria de niños con PC.

Tras obtener la aprobación del comité de ética, se solicitó una lista de todos los niños con PC atendidos en consulta médica en el SRP-HNERM durante el año 2024. Posteriormente, se revisaron manualmente 86 historias clínicas electrónicas para extraer la información relevante en una hoja de cálculo de Microsoft Excel.

Se consideraron variables sobre características generales, características funcionales y clínicas, relacionadas con la necesidad (según evaluación del fisiatra en la consulta externa, basada en la historia clínica, el examen físico y el desempeño funcional del paciente) y la utilización previa de servicios de rehabilitación (antecedente de recepción de intervenciones fisiátricas para manejo de la espasticidad y terapias de rehabilitación), uso de ortesis o dispositivos de asistencia, y tratamiento farmacológico, las cuales se detallan en una tabla de operacionalización de variables (material suplementario).

Los datos recolectados en la hoja de cálculo fueron importados al programa Stata versión 18.0 (StataCorp LLC, College Station, Texas, Estados Unidos). Las variables numéricas se reportaron como medias y desviaciones estándar o medianas y rangos intercuartílicos, en función a su normalidad evaluada mediante la prueba de Shapiro-Wilk. Las variables categóricas se presentaron como frecuencias absolutas y relativas.

Este estudio fue aprobado por el Comité Institucional de Ética en Investigación del HNERM (Certificado de Calificación Ética AUT N.º 095-CE-GHNERM-GRPR-ESSALUD-2024). Toda la información recolectada se mantuvo estrictamente confidencial y fue utilizada exclusivamente para los fines del estudio.

## HALLAZGOS

Se evaluaron 86 niños con PC. La mediana de edad fue de nueve años. La mayoría eran varones (57%), residían en Lima (74,4%), eran pacientes continuadores (72,1%), tenían bajo peso (44,2%), y no tenían antecedente de prematuridad (61,6%). El 47,7% presentó discapacidad intelectual severa/profunda, el 18,6% no tenía evaluación de coeficiente intelectual, y el 41,8% no asistía a ninguna institución educativa. El 44,2% no contaba con certificado de discapacidad y, entre quienes lo tenían, el 46,5% fue calificado con gravedad severa, con una media de tiempo para su obtención de 4,86 años desde el diagnóstico de PC (tabla 1).

**Tabla 1:** Características generales de los niños con parálisis cerebral atendidos en el Servicio de Rehabilitación Pediátrica del Hospital Nacional Edgardo Rebagliati Martins en Lima, Perú, durante el 2024 (n=86).

Variable		n (%)
Edad en años		9 (6-11)^a^
Sexo		
	Masculino	49 (57,0)
	Femenino	37 (43,0)
Domicilio		
	Lima	64 (74,4)
	Fuera de Lima	22 (25,6)
Tipo de paciente		
	Continuador	62 (72,1)
	Nuevo	24 (27,9)
Índice de masa corporal		
	Normal	40 (46,5)
	Bajo peso	38 (44,2)
	Sobrepeso	8 (9,3)
Antecedente de prematuridad		
	No	53 (61,6)
	Prematuro moderado a tardío	13 (15,1)
	Muy prematuro	5 (5,8)
	Prematuro extremo	15 (17,5)
Discapacidad intelectual		
	Severa/profunda	41 (47,7)
	Leve	5 (5,8)
	No	24 (27,9)
	Sin evaluación	16 (18,6)
Educación		
	Ninguna	36 (41,8)
	Básica regular	35 (40,7)
	CEBE	14 (16,3)
	PRITE	1 (1,2)
Tenía certificado de discapacidad		48 (55,8)
	Gravedad leve	3 (3,5)
	Gravedad moderada	5 (5,8)
	Gravedad severa	40 (46,5)
Tiempo en meses sin certificado de discapacidad desde el diagnóstico de PC (n=38)		4,86 (3,22)^b^

CEBE: Centro de Educación Básica Especial, PRITE: Programa de Intervención Temprana, PC: parálisis cerebral.

a Mediana (rango intercuartílico), b media (desviación estándar).

La mayoría presentó hemiparesia espástica (37,2%) o cuadriparesia espástica (27,9%), así como niveles altos de discapacidad funcional (Sistema de la Clasificación de la Función Motora Gruesa [GMFCS] V, Sistema de Clasificación de la Habilidad Manual [MACS] V, Sistema de Clasificación de la Habilidad para Comer y Beber [EDACS] III-IV). Las comorbilidades más frecuentes fueron epilepsia (53,5%) y luxación de cadera (23,3%). Solo el 8,1% tenía gastrostomía y el 47,7% contaba con una radiografía de pelvis. La mayoría presentó pérdida de rango articular de movimiento (RAM) pasivo en una o más articulaciones (73,3%) (tabla 2). En relación con el tono muscular, el hemicuerpo izquierdo fue el más afectado (figura 1).

**Tabla 2:** Características funcionales y clínicas de los niños con parálisis cerebral atendidos en el Servicio de Rehabilitación Pediátrica del Hospital Nacional Edgardo Rebagliati Martins en Lima, Perú, durante el 2024 (n=86).

Característica		n (%)
Clasificación por subtipos		
	Hemiparesia espástica	32 (37,2)
	Cuadriparesia espástica	24 (27,9)
	Paraparesia espástica	10 (11,6)
	Cuadriplejía espástica	8 (9,3)
	Paraplejía espástica	1 (1,2)
	Otra	11 (12,8)
GMFCS		
	V	36 (41,8)
	IV	8 (9,3)
	III	4 (4,7)
	II	27 (31,4)
	I	11 (12,8)
MACS		
	V	26 (30,2)
	IV	14 (16,3)
	III	11 (12,8)
	II	20 (23,3)
	I	15 (17,4)
EDACS		
	V	9 (10,5)
	IV	21 (24,4)
	III	21 (24,4)
	II	16 (18,6)
	I	19 (22,1)
Comorbilidades		
	Epilepsia	46 (53,5)
	Luxación de cadera	20 (23,3)
	Hidrocefalia	14 (16,3)
	Pie equinovaro	12 (13,9)
	Escoliosis	11 (12,8)
	Enfermedad respiratoria	10 (11,6)
Tenía gastrostomía		7 (8,1)
Tenía radiografía de pelvis		41 (47,7)
Tenía pérdida del RAM pasivo en una o más articulaciones		63 (73,3)

GMFCS: Sistema de Clasificación de la Función Motora Gruesa, MACS: Sistema de Clasificación de la Habilidad Manual, EDACS: Sistema de Clasificación de la Habilidad para Comer y Beber, RAM: rango articular de movimiento.

**Figura 1:** Tono muscular (media de la puntuación en la Escala Modificada de Ashworth) por extremidad en niños con parálisis cerebral (n=86).

Respecto a la necesidad y utilización previa de servicios de rehabilitación, aunque el 55,8% necesitaba toxina botulínica tipo A (BoNT-A) para el manejo de la espasticidad, la mayoría no la había recibido previamente (58,3%). El 68,6% requería bloqueos nerviosos diagnósticos. Casi todos requerían terapia física (TF) (99,8%), ocupacional (TO) (90,7%) y de lenguaje/deglución (TL) (75,6%); no obstante, solo el 54,1% había recibido TF previamente, mientras que la mayoría no había recibido TO (85,9%) ni TL (81,5%). Asimismo, el 19,8% interrumpió sus terapias de rehabilitación entre uno a cuatro años debido a la pandemia de COVID-19, con una media de tiempo de interrupción de 2,4 años (tabla 3).

**Tabla 3:** Necesidad y utilización de servicios de rehabilitación, uso de ortesis o dispositivos de asistencia, y tratamiento farmacológico de niños con parálisis cerebral atendidos en el Servicio de Rehabilitación Pediátrica del Hospital Nacional Edgardo Rebagliati Martins en Lima, Perú, durante el 2024 (n=86).

Característica		n (%)
Necesidad de DNB para manejo de la espasticidad		59 (68,6)
Necesidad de BoNT-A para manejo de la espasticidad (n=48)		
	Recibió previamente BoNT-A	20 (41,7)
	Nunca recibió BoNT-A	28 (58,3)
Necesidad de TF (n=85)		
	Recibió previamente TF	46 (54,1)
	Nunca recibió TF	39 (45,9)
Necesidad de TO (n=78)		
	Recibió previamente TO	11 (14,1)
	Nunca recibió TO	67 (85,9)
Necesidad de TL (n=65)		
	Recibió previamente TL	12 (18,5)
	Nunca recibió TL	53 (81,5)
Tiempo en años transcurrido sin terapias de rehabilitación (n=17)		
	1 año	2 (11,7)
	2 años	7 (41,2)
	3 años	7 (41,2)
	4 años	1 (5,9)
Media del tiempo en años sin terapias de rehabilitación^a^		2,41 (0,79)^b^
Uso de ortesis y dispositivos de asistencia		74 (86,0)
	Ortesis tobillo-pie	60 (69,8)
	Silla de ruedas/coche adaptado	40 (46,5)
	Ortesis larga de rodilla	30 (34,9)
	Férula de muñeca-mano	27 (31,4)
	Ortesis estática de codo	18 (20,9)
	Órtesis toracolumbar semirrígida	11 (12,8)
	Andador	5 (5,8)
Uso de anticonvulsivantes		46 (53,5)
	Uno	21 (24,4)
	Dos	14 (16,3)
	Tres	11 (12,8)
Uso de baclofeno		26 (30,2)
Uso de broncodilatadores		9 (10,5)
Use de cannabinoides		3 (3,5)

DNB: bloqueo nervioso diagnóstico; BoNT-A: toxina botulínica tipo A; TF: terapia física; TO: terapia ocupacional; TL: terapia de lenguaje/deglución.

a Tiempo transcurrido sin terapias de rehabilitación debido a la pandemia de COVID-19, desde la última sesión de terapia, ^b^ media (desviación estándar)

El 86% utilizaba ortesis o dispositivos de asistencia, siendo los más frecuentes las ortesis tobillo-pie (OTP) (69,8%), la silla de ruedas o coche adaptado (46,5%) y la ortesis larga de rodilla (34,9%). En cuanto a tratamiento farmacológico, el 53,5% utilizaba anticonvulsivantes, y el 30,2% baclofeno (tabla 3).

## DISCUSIÓN

La mayoría de los niños con PC tenía bajo peso. El 47,7% presentó discapacidad intelectual severa/profunda y el 41,8% no asistía a ninguna institución educativa. Poco menos de la mitad no contaba con certificado de discapacidad y, entre quienes lo tenían, la media de tiempo para su obtención fue de 4,86 años. La luxación de cadera fue una de las comorbilidades más frecuentes, pero menos de la mitad contaba con una radiografía de pelvis. El 45,9%, 85,9% y 81,5% de quienes requerían TF, TO y TL, respectivamente, nunca las habían recibido, pese a la alta frecuencia de limitaciones moderadas a severas en función motora gruesa (GMFCS V), manipulación (MACS V) y deglución (EDACS III-IV), incluyendo uso de gastrostomía.

Una revisión sistemática publicada en el 2022 reportó una prevalencia de malnutrición cercana al 40% en niños con PC, asociada a escasez de alimentos y factores socioeconómicos y etarios ^11^. La prevalencia observada en este estudio podría explicarse por hallazgos del Registro Global de PC en LMICs, que reportó mayores tasas de malnutrición asociadas a menor educación materna y mayor compromiso motor (GMFCS III-V) ^12^. Cabe destacar que el peso/edad es la medida antropométrica más utilizada para evaluar el estado nutricional ^11^; sin embargo, en el SRP-HNERM se empleó el IMC.

El GBD 2019 reportó alta prevalencia de discapacidad intelectual en pacientes con PC, y resaltó la importancia de la identificación e intervención temprana, especialmente en LMICs, para mejorar la preparación escolar y promover la educación inclusiva ^7^. En Perú, desde el 2024, una ley promueve la capacitación de profesionales de salud y docentes en educación inclusiva para niños con PC ^13^. Un estudio de cohorte en Australia encontró que solo el 30% de los niños con PC presentaba puntajes normales en pruebas cognitivas, teniendo la mayoría hemiplejia o hemiparesia ^14^, lo cual coincide con los hallazgos de este estudio.

En Perú, la Ley General de la Persona con Discapacidad establece que la certificación acredita la condición de persona con discapacidad y otorga beneficios sociales y laborales ^15^; sin embargo, este estudio halló una prolongada media de tiempo para su obtención, que podría relacionarse con la media de edad de diagnóstico de PC reportada previamente (4,1 años) ^5^. Estudios en la región han identificado barreras para la certificación de la discapacidad, como desconocimiento médico, limitaciones tecnológicas y dificultades logísticas, incluyendo acceso limitado a Internet y residencia en zonas rurales ^16^.

Los programas establecidos de vigilancia de cadera para niños con PC recomiendan una evaluación clínica y radiográfica entre el primer y segundo año o desde el diagnóstico, para una detección y tratamiento tempranos ^17^. Los pacientes con GMFCS V presentan un riesgo de luxación de cadera de hasta 90%, condición que afecta significativamente la función y la calidad de vida, al limitar la sedestación, la movilidad y las actividades diarias, y constituir una causa frecuente de dolor ^17^.

Un estudio transversal realizado en 2023 en niños con PC atendidos en su mayoría en hospitales públicos de Arabia Saudita encontró que el 96,4% había recibido TF, pero que el 65,9% y el 85,7% no habían recibido TO y TL, respectivamente ^18^. Un estudio transversal realizado en 2025 en niños con PC atendidos en centros de rehabilitación de Argentina encontró que todos habían recibido al menos una hora semanal de terapias de rehabilitación ^19^. Las diferencias con el presente estudio podrían explicarse por el bajo número de fisiatras y terapeutas de rehabilitación en el SRP-HNERM para la atención de esta población. Además, un estudio del 2016 en niños peruanos con PC encontró una mediana de tiempo de espera de dos meses para recibir TF ^7^. Las personas con discapacidad enfrentan, además, otras barreras para acceder a los servicios de rehabilitación, como la falta de conocimiento de las familias sobre su importancia y las limitaciones en el acceso al transporte ^19^.

Este estudio presenta algunas limitaciones que deben considerarse al interpretar sus resultados. En primer lugar, al tratarse de un estudio retrospectivo basado en historias clínicas, existe la posibilidad de errores de registro y de información incompleta para algunas variables no sistematizadas en la práctica clínica habitual. En segundo lugar, el estudio incluye únicamente niños con PC atendidos en el SRP-HNERM, lo que introduce un importante sesgo de selección, ya que esta población no es representativa de todos los niños con PC en el Perú, particularmente de aquellos atendidos en establecimientos del Ministerio de Salud (MINSA) del Perú o de quienes no acceden a ningún nivel de atención. En estos grupos, la utilización de servicios de rehabilitación podría ser menor o incluso inexistente, por lo que los hallazgos podrían subestimar o sobreestimar ciertas brechas en otros contextos asistenciales. Finalmente, debido a su diseño transversal y unicéntrico, los resultados no permiten establecer inferencias causales ni son generalizables a la población total de niños con PC del país. Sin embargo, este estudio aporta evidencia detallada de un grupo atendido en un centro de referencia, lo que permite describir patrones de necesidad y utilización en un entorno con disponibilidad relativa de servicios de rehabilitación.

En conclusión, se evidenció una importante discrepancia entre la necesidad y la utilización previa de servicios de rehabilitación en los niños con PC evaluados, especialmente en terapia ocupacional, terapia de lenguaje/deglución y manejo de la espasticidad, así como en la vigilancia radiográfica de cadera y la obtención del certificado de discapacidad. Esta situación se presentó en el contexto de una población con alta carga de compromiso funcional y comorbilidades, incluyendo bajo peso, discapacidad intelectual severa/profunda y limitaciones significativas en la función motora y de la alimentación. En conjunto, estos hallazgos aportan evidencia descriptiva relevante para la comprensión de la brecha entre la necesidad y la utilización de servicios de rehabilitación en este contexto clínico.
